# Brain Process for Perception of the “Out of the Body” Tactile Illusion for Virtual Object Interaction

**DOI:** 10.3390/s150407913

**Published:** 2015-04-01

**Authors:** Hye Jin Lee, Jaedong Lee, Chi Jung Kim, Gerard J. Kim, Eun-Soo Kim, Mincheol Whang

**Affiliations:** 1 Department of Emotion Engineering, Graduate School, Sangmyung University, 7 Hongji-dong, Jongro-Ku, Seoul 110-743, Korea; E-Mails: hyejinne@gmail.com (H.J.L.); gatsgrain@nate.com (C.J.K.); 2 College of Information and Communications, Korea University, Anam-dong 5-ga, Seongbuk-gu, Seoul 136-791, Korea; E-Mails: jdlee@korea.ac.kr (J.L.); gjkim@korea.ac.kr (G.J.K.); 3 HoloDigilog Human Media Research Center (HoloDigilog), 3D Research Center (3DRC), Kwangwoon University, 447-1Wolge-Dong, Nowon-Gu, Seoul 139-701, Korea; E-Mail: eskim@kw.ac.kr; 4 Department of Media Software, Sangmyung University, 7 Hongji-dong, Jongro-Ku, Seoul 110-743, Korea

**Keywords:** phantom sensation, illusory feedback, funneling, vibro-tactile feedback, neural mechanism, EEG, ERP

## Abstract

“Out of the body” tactile illusion refers to the phenomenon in which one can perceive tactility as if emanating from a location external to the body without any stimulator present there. Taking advantage of such a tactile illusion is one way to provide and realize richer interaction feedback without employing and placing actuators directly at all stimulation target points. However, to further explore its potential, it is important to better understand the underlying physiological and neural mechanism. As such, we measured the brain wave patterns during such tactile illusion and mapped out the corresponding brain activation areas. Participants were given stimulations at different levels with the intention to create veridical (*i.e.*, non-illusory) and phantom sensations at different locations along an external hand-held virtual ruler. The experimental data and analysis indicate that both veridical and illusory sensations involve, among others, the parietal lobe, one of the most important components in the tactile information pathway. In addition, we found that as for the illusory sensation, there is an additional processing resulting in the delay for the ERP (event-related potential) and involvement by the limbic lobe. These point to regarding illusion as a memory and recognition task as a possible explanation. The present study demonstrated some basic understanding; how humans process “virtual” objects and the way associated tactile illusion is generated will be valuable for HCI (Human-Computer Interaction).

## 1. Introduction

Vibro-tactile stimulation is used popularly as an inexpensive, but effective sensory feedback for enhancing human computer interaction experience. The most usual form of the application of vibro-tactile stimulation is by using a single vibrator embedded in a mobile device. This form of stimulation is limited in providing rich interaction feedback aside from simple on-off type of events. Using a tactile grid [1,2,3,4] with many vibrators, on the other hand, as an alternative, is a difficult proposition due to cost and usability (e.g., a large area of the body has to be in full contact with the grid, which makes it difficult and cumbersome to use).

**Figure 1:**
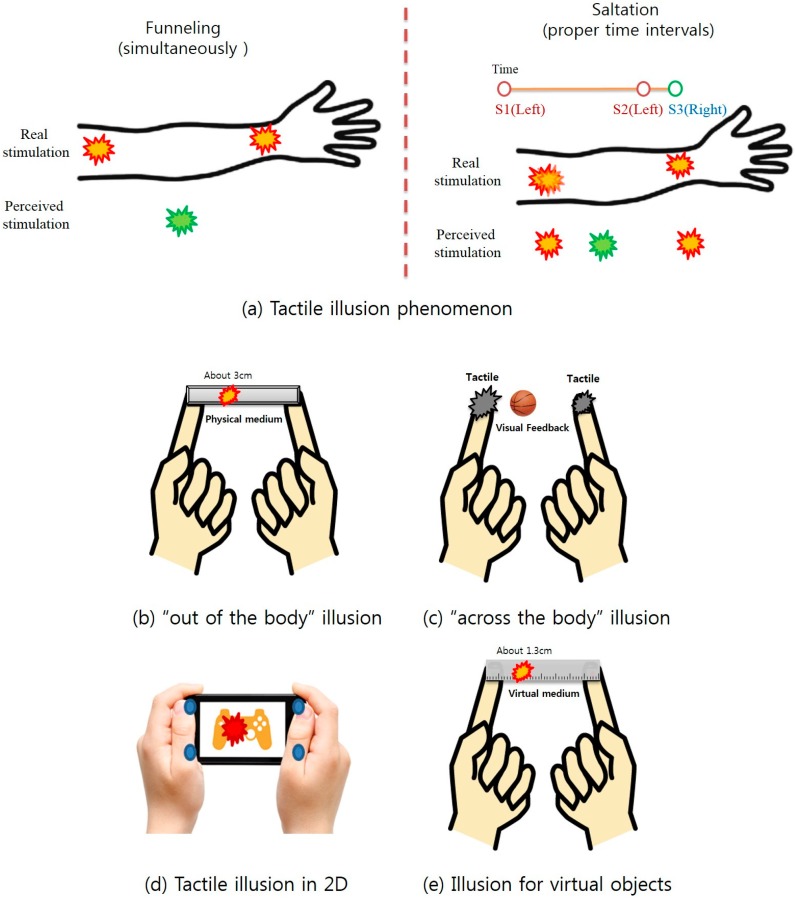
(**a**) Illustration of the two main illusory tactile sensations (funneling and saltation) [5,6] and recent extensions: (**b**) “out of the body” illusion (tactile experience from a hand-held physical medium) [10]; (**c**) “across the body” illusion (e.g., between hands of separate limbs) [11]; (**d**) 2D modulation for hand-held mobile interaction [12]; and (**e**) illusion for augmented object (tactile interaction using both tactile illusion (funneling/saltation) and virtual visual feedback [14]).

Recently, tactile illusion has emerged as a viable solution to this problem, because it can induce “phantom” vibro-tactile sensation as if emanating from a location that is not in direct contact with the actuators. For instance, saltation [5,6] elicits pseudo-tactile sensations between two locations on the body by presenting stimuli at the two locations with a proper time delay. Another major tactile illusion technique, called funneling [7,8,9], can elicit similar phantom sensation, simultaneously stimulating the two skin locations with different amplitudes (see Figure 1). In addition, several extensions to these tactile illusions have been discovered or developed, such as the “out of the body” phenomenon (phantom sensation from an external hand-held body-mediating object) [10], “across the body” saltation (saltation on a non-continuous body, e.g., between hands of separate limbs, such as right and left arms/fingers) [11], the 2D modulation technique (using four stimuli for modulating tactile illusion in 2D) in a mobile device [12,13] and illusion for virtual objects (phantom sensation from augmented objects) [14] (also see Figure 1). Despite these developments, not much work has been done in investigating the underlying physiological and neural mechanism. Such a mechanism is very important and would be critical in understanding how these tactile illusion techniques can be further applied to and extended for human computer interaction in a safe and effective manner. Note that such illusory tactile sensation can be implemented with only a small number of vibrators (e.g., compared to a tactile grid), while generating a richer tactile experience than the simple single vibrator scheme.

**Figure 2:**
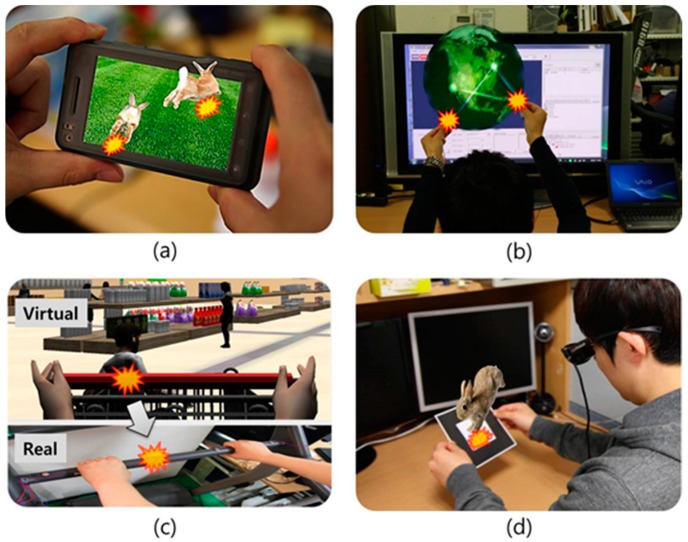
Possible applications of the “out of the body” phantom tactile sensation: two-handed/fingered interaction and feeling tactile sensations as if coming from the middle of: (**a**) a mobile device; (**b**) holographic imagery; (**c**) indirectly from a virtual object in a monitor; and (**d**) an augmented marker (e.g., seen through a head-mounted display).

Thus, in this paper, we investigate the brain activation patterns associated with funneling (one of the major tactile illusion phenomenon) for a virtually-augmented object using brain waves (EEG/electroencephalography). Among many varieties of tactile illusion, this was chosen because it represents an interesting case; the illusion is produced for a “virtual” (without any direct tactility or physicality) object external to the body. For example, will the theoretical explanation of the illusion through the body map (*i.e.*, cortical homunculus) extension in the brain (as referred to in the Section 2) also apply to virtual objects? Few brain image studies have been conducted for basic tactile stimulations and for the illusion elicited directly on the skin [8,15,16,17]. From these studies, we do have some basic understanding of the sensory information processing pathways in the brain, and this can serve as a reference for our study. How humans process “virtual” objects and the way associated illusory tactile illusion is generated will be valuable for HCI, more specifically for virtual/mixed reality, multimodal games and mobile interaction (see Figure 2).

In this study, EEG measurement has been used to look for any differences in the brain activation when illusory tactile sensation (as generated by the funneling technique) was felt with an “out of the body” virtual object (in an augmented reality setting) as compared to when a veridical sensation was felt. In particular, we focused on the ERP (event-related potential) response between the illusory and veridical sensations. Furthermore, sLORETA (standard low resolution brain electromagnetic tomography) was also applied to locate the signal sources of the brain activation areas. We have observed P300 latency and amplitude and the activation region per frequency band according to stimulus with sLORETA software. P300 is known to be associated with complex cognitive function, rather than tactile sensory processing (for details, review [18]). Illusory information processing was reported to be affected by cognitive response [19]. The activation region per frequency band was observed, as sustainable task performance has been known to influence all of the frequency bands of EEG due to attention and memory [20,21,22,23,24]. fMRI (functional magnetic resonance imaging) was not used, due to the operational difficulty with the augmented reality setting and vibratory stimulation within the core (see later sections for more details). 

In the next section, we first review previous research literature related to phantom tactile sensation and its application to practical interaction design. Then, we describe the validation experiment for the “out of the body” funneling and report the results. Finally, we conclude the paper with a discussion and directions for future research.

## 2. Related Work

Phantom sensation generally refers to the phenomenon that user’s perception does not match the stimulus’ physical characteristics. Funneling, along with saltation, is one of the two major perceptual illusion techniques for vibro-tactile feedback. It refers to stimulating the skin at two (or more) different locations simultaneously with different amplitudes and eliciting phantom sensations in the space between [7,25]. Several researchers have applied this phenomenon to human interfaces, experimenting with different ways of modulating the vibration amplitudes for detailed controlling of the target phantom sensation locations [7,26,27]. Mizukami and Sawada [28] have used funneling to generate phantom sensations in 2D and applied it to tactile character recognition.

Recently, Miyazaki has discovered that tactile illusion can be extended to body-worn (e.g., hand-held) objects and can create “out of the body” tactile experience [10].“Out of the body” tactile experience refers to the phenomenon in which one can perceive tactility as if emanating from a location external to the body without any stimulator placed at the same location where the illusion is perceived. As shown in Figure 1, in order for the out of the body illusion to happen, the external object must visually connect two parts of the body (e.g., the two finger tips).Moreover, Lee *et al.* have found that this phenomenon carried over to virtual objects, as well [14].This form of tactile illusion is appealing in its practical application to HCI, because the interaction scheme imposes less physical constraints between the device and user’s body. For example, if a usual 2D array of tactile actuators is used, a significant part of the user body (e.g., the whole palm) would have to be in constant contact with the array to receive any feedback. In another similar case of the tactile grid chair [29], the user needs to maintain tight contact with his or her back to the tactile array, making it difficult to use, thus limiting its practical application. 

The body map in the brain (cortical homunculus) is often cited as one plausible explanation for these illusion phenomena. Ramachandran *et al.* [30] studied the phantom limb (rubber hand illusion) phenomenon using the EEG signals and offered the theory of a “dynamically reconfiguring” body map, and a few studies do relate the encodings in the body map to saltation or funneling [8,15,16,17].There are other cognitive explanations, as well [31,32].For example, it seems plausible to hypothesize that the physical connection of the external object to the body induces the body map reconfiguration (*i.e.*, to treat the external object as part of one’s body). However, as for the out of the body illusion for “virtual” objects, the connection is only visual or virtual (not physical), and thus, the underlying mechanism remains as an interesting scientific issue. Ehrsson *et al.* also studied the brain mechanism for the rubber hand illusion and associated the sense of ownership with the parts of the brain responsible for multisensory fusion rather than just the visual cortex [33]. Slater *et al.* [34] and Sanchez-Vives [35] both studied virtual hand illusions; however, they did not offer a brain scientific explanations for them. Rubber/virtual hand illusion and illusion in this study are similar in that both are elicited in mediated environments using vibrotactile stimulations [36]. However, the tactile illusion in this study was based on feedback that was felt outside the subject’s body from interaction with a visual object. This is different from the rubber hand illusion case, in which subjects’ were confused about whether the tactile illusion was happening on their body.

Few scientists have used brain imaging, such as fMRI, to study illusions, such as saltation. Chen *et al.* [8] showed with optical imaging with simultaneous stimulation of two fingertips (using funneling) produced a single focal cortical activation between the single fingertip activation regions. Furthermore, he found that the amplitudes of activation produced by paired digit stimulation (both adjacent and non-adjacent) were smaller than the sum of the single-digit activation areas. Blankenburg *et al.* [37] have found brain activation on the premotor and prefrontal cortex area using fMRI, in the condition of a tactile illusion on the forearm. He compared the illusory (classic cutaneous rabbit illusion) and veridical stimulation of the forearm and found that illusory percepts can affect primary somatosensory cortex in a manner equivalent to physical stimulation. Similarly, Gross [17] discovered that the same brain sector was activated regardless of whether the tactile sensation is illusory or veridical (using a 3T fMRI). These results have reassured the statement regarding the relationship between illusory tactile sensation and primary somatosensory cortex. Tactile illusion can affect primary somatosensory cortex in a manner that corresponds “somatotopically” to the phantom sensation. However, the out of body tactile illusion is still unexplored and not wholly understood. As the “out of body” tactile illusion generates an illusory external sensation by imposing actual stimulation on participants’ body, the limbic area, where memory recall occurs, is expected to be activated [38].

## 3. Experimental Section

**Figure 3:**
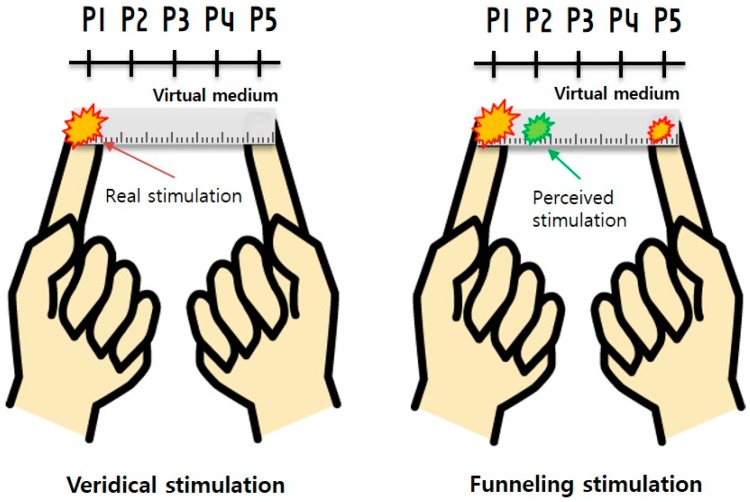
The two conditions in the experiment: veridical stimulations on the fingertips at P1 and P5 (note that P1 and P5 are where the vibrators are actually located) and funneling stimulation to induce illusory sensation at P2–P4 with the virtual object seen to connect the body.

In this experiment, we measure and compare the subject’s EEG responses to two types of tactile stimulations, that is: (1) veridical stimulations on the two finger tips at P1 and P5 (reference response); and (2) funneling stimulation to elicit illusory sensation at P2–P4 using a virtual object that connects the two finger tips by visual augmentation. Figure 3 shows the experimental set up. The ultimate objective is to analyze the brain activities, identify their source areas and offer a neural explanation to the tactile illusion of the out of the body virtual object.

### 3.1. Experimental Setup

**Figure 4:**
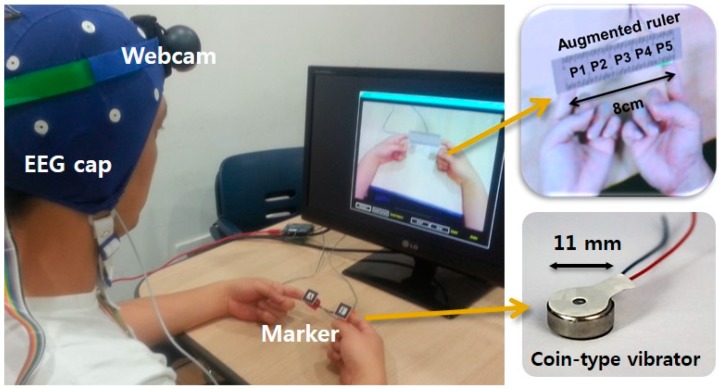
The experimental set up for experiment. Vibratory stimulations were given to the two index fingers, and a user watched the monitor where an augmented virtual ruler was placed on the fingers. An EEG cap was used to measure and document the brain activity.

The users were given simultaneous tactile stimulations to their two index fingers, one on the right and the other on the left. The two fingertips were tracked using small markers by a head-mounted camera, and an augmented reality video imagery was presented to the user as visual feedback in which a virtual ruler was overlaid (to look as if being) in between the two fingers (see Figure 4). The small markers were attached to the respective fingers using tape, and the users were allowed to freely move or twist the fingers to some extent. AR Toolkit [39] was used to recognize and track the small markers and to generate the augmented video imagery. A webcam was worn on the head-mounted fixture to produce a view close to one according to the actual line of sight. This set up was used to generate a natural feel of “holding” the object, by allowing small movements of the hands/fingers and still maintaining natural augmented imagery. The holding distance for the virtual ruler (augmented imagery) was kept at 8 cm by asking the user to maintain the finger positions stably. The ruler stayed augmented, even if the fingers became separated by more than 8 cm, but only to a limited degree (±2.5 cm). There was virtually no operational difficulty in this regard. The visual length of the virtual ruler was calibrated to match the physical units.

In addition to the head-mounted fixture for the camera, the participants wore ear muffs to prevent any bias from the sounds of the vibration. The ear muff was tested to make sure that no sound could be heard during the experiment and did not affect the outcome of the experiment.

**Figure 5:**
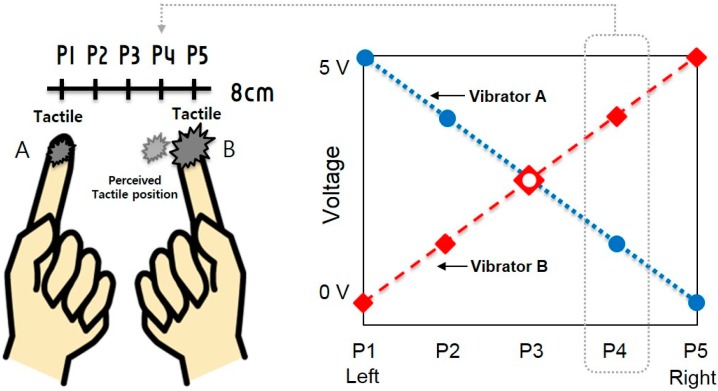
The amplitude rendering (as originally proposed by Alles [7]) for funneling to create phantom (or real) sensations at five different positions (P1–P5). For example, to generate a phantom sensation at P4, Vibrator A’s amplitude is set at 1.25 V and Vibrator B 3.75 V.

The stimulations were given using two vibration motors, whose respective amplitudes were chosen by an interpolation function intended to create phantom tactile sensations at particular locations, and the user was asked where on the virtual ruler he or she felt the tactile feedback. The linear variation of the stimulus amplitude methods of Alles [7] was used to create and modulate the locations of the out of body tactile illusion, as shown in Figure 5. In the linear rendering method (proposed by Alles), the amplitude of one tactile stimulation was linearly increased, while that of the other tactile stimulation was decreased. 

A common coin-type vibrator was used (placed on the respective fingertips) and controlled by an Arduino board [40] (and interfaced to and synchronized with the AR Toolkit-based experiment software). The vibration motor used in our experiment has the same specification [41] as reported in [42]: flat/circular and sized 11 mm in diameter (see Figure 2). It is controlled by a voltage input using a pulse width modulation signal with an amplitude between 0 to 5 V, which, in turn, produces vibrations with a frequency between 0 and 250 Hz and associated amplitudes between 0 to 2 g. (measured in acceleration, or 0 to 18 μm in position), respectively. According to [42,43,44], these values are well above the human’s normal detection threshold (about 6–45 dB).

An EEG cap was used to measure and document the brain activity. EEG data were attained at the 19 electrode sites (Fp1, Fp2, F7, F3, Fz, F4, F8, T3, C3, Cz, C4, T4, T5, P3, Pz, P4, T6, O1 and O2) on the EEG cap (BIOPAC CAP100C, BIOPAC Systems, Goleta, CA, USA) of the international 10–20 system [45]. All of these electrodes had an impedance of 5 kΩ, and data were sampled with a frequency of 250 Hz. The low-cut and high-cut filters were each set to 0.3 Hz and 50 Hz, respectively. Furthermore, the notch filter was set at 55–65 Hz. The EEG data were recorded with the WinEEG software Version 2.84.44 (Mitsar, St. Petersburg, Russia) and the EEG amplifier (Mitsar EEG 201, St. Petersburg, Russia).

### 3.2. Experimental Task and Detailed Procedure

Eighteen university students between 23 and 32 years of age (10 females and 8 males, mean age: 24) participated in the experiment. All participants were right-hand correlated and had no history of any disorder that would impede the process of gathering brain activity using EEG. In order to induce their full attention and active cooperation, proper monetary compensation was given to each subject. In addition, on the day prior to the experiment, subjects were instructed to restrict themselves from consuming any alcohol and caffeine, as well as smoking. Subjects were also asked to take a full sleep the previous night.

Training was given for the participant to get familiarized with the experimental process. The training consisted of one series of 10 trials for each stimuli (5 stimuli). Each trial consisted of stimulation (200 ms) and an inter-stimulus interval (3 s). The training set lasted for a total of 160 s. Participants were asked about their knowledge of tactile illusion, and all answered that they had neither the knowledge nor the experience of it. For each subject, the non-stimulation reference EEG signals were first captured and recorded.

The participants were made to sit comfortably on the chair and were informed about the process and purpose of the experiment. Participants were instructed to not move. Furthermore, the participant was asked to refrain from blinking as much as possible for intact EEG signals. Although the WinEEG program that we used to collect PCA components from raw EEG data removes eye blinking artifacts, the authors were trying to reduce the noise due to eye blinking as much as possible to have cleaner data.

The levels of stimulations were given with the intention of creating veridical or phantom sensations at five different points along the ruler (note that the stimulations at the two extreme ends of the ruler are real). In the veridical condition, the tactile stimulation was given only to one finger tip, at P1 or P5. This condition merely serves as a reference matching to the brain activations for real tactile sensation. In the phantom condition, tactile stimulation was given to two fingertips with interpolated values in order to elicit illusions, not only at the fingertips themselves (P1 and P5), but also at the intermediate positions, at P2–P4.The virtual ruler was augmented to appear as 10 cm with the two fingers holding it at 1 cm from the two ends (at the1 cm and 9 cm marks with the inter-stimulus distance = 8 cm). The inter-finger distance of 8 cm was set equal to the experimental condition used in [10].

The previous study also confirmed that the aforementioned linear method for administrating the funneling-based stimulations exhibited the best performance in terms of localization performance and reliability [14]. The within group design was employed for the experiment. On a single experiment for each participant, a block of 100 positional feedbacks, which consisted of 3 s of an inter-stimulus rest interval and a 200-ms stimulation duration, was suggested. Five hundred trials per experiment were held. Stimulus was given on the P1 to P5 positions with balanced order to randomize the presentation. In order to avoid pitfalls of standard repeated measure designs, experimental conditions were counterbalanced. The EEG signal was measured while the stimulus was present to observe the participants’ brain activations for the given stimulus. The total duration of the experiment was 1600 s, which was completed in a single day. 

To minimize external noise, the experiment was held in an enclosed room with soundproofing. The size of the room was 2.87 × 3.62 m, and light intensity was maintained at120 lux. Three participants per day conducted the experiment on 10:00, 14:00 and 17:00, Korean standard time.

After all trials of the experiment, participants answered a questionnaire on four categories: tactile illusion, localization, recognition time and level of certainty on their response, all answered in the 7-point Likert scale (see Table 1).

**Table 1:** Four questions regarding the subjective feeling of the tactile sensation responded in a 7-point Likert scale.

Number	Question
Q1	Were you able to perceive a sensation from an empty space between the two index fingers? (1: not at all ~7: very well)
Q2	Could you perceive the location of stimulation? (1: not at all ~7: very well)
Q3	How long did it take you to perceive the tactile feedback, if any? (1: instantly ~4: 1–2 s ~7: more than 3 sec)
Q4	How certain are you about your answer overall to Q1, Q2 and Q3? (1: not certain at all ~7: very certain)

## 4. Analysis Methods 

The raw EEG data were pre-processed to remove artifacts using the PCA (principal component analysis) methods available from the WinEEG package (Mitsar, St. Petersburg, Russia), the software package used for the analysis. PCA was used to remove the artifacts and noise of the EEG. This method uses orthogonal transformation to convert the observation matrix. The principal components, which are uncorrelated variables, will show normality with other variables. These orthogonal variables are selected and linearly combined to reconstruct the raw data into more useful data [46].

To carry out the ERP analysis with the pre-processed EEG data and the EEG data, both were categorized according to five different lobes, which are frontal, temporal, central, parietal and occipital lobe (see Table 2), and to the locations of veridical and phantom sensation (see Figure 3). Note that P1 and P5 correspond to the fingertip locations (where veridical sensation would be felt) and P2–P4 the intended locations of the phantom sensation. Each lobe of the brain is associated with specific characteristic activity. Frontal lobe is associated with cognitive and reasoning function, while parietal lobe organizes touch sense. Occipital lobe, on the other hand, is in charge of visual information processing [47]. Therefore, in the current study, to identify the ROI signal related to tactile illusion, electrodes were categorized accordingly to each lobe, as in Table 2. The electrode clustering method was taken from [48,49]. For each category, a grand-mean was obtained across 18 participants. For ERP quantification, peak amplitude and latency values were determined with the automated process of the maximum peak amplitude analysis [50,51] and confirmed by visual inspection using the following time windows: P300 (200–400 ms). These amplitude and latency averages were found for all 19 receptor channels (or the five lobes of the brain). Such data collection and processing were done for each treatment, namely for when a tactile illusion was induced and when veridical stimulation was given.

**Table 2:** Categories of EEG data according to each lobe for analysis.

Categories of EEG Electrodes (Lobe)	Electrodes
Frontal lobe	Fp1, Fp2, F7, F3, Fz, F4, F8
Temporal lobe	T3, T4, T5, T6
Central lobe	C3, Cz, C4
Parietal lobe	P3, Pz, P4
Occipital lobe	O1, O2

The averages of the amplitude and latency for the two conditions were compared using two types of statistical tests: (1) Mann–Whitney U test; and (2) Kruskal–Wallis test. The non-parametrical test was adopted as normality verification test with data rejecting the null hypothesis. As for the Mann–Whitney U test, two groups of data were compared: between the veridical (at P1 and P5) and illusory (at P2, P3 and P4). For the Kruskal-Wallis test, three groups of data were compared: among the veridical (at P1 and P5), close-illusory (responses at P2 and P4) and far-illusory (responses at P3), to validate the following hypotheses:

(1) There exist significant differences in ERP responses between the veridical and illusory conditions (Mann–Whitney U test/Kruskal–Wallis test).
(2) There exist significant differences in ERP responses among the veridical, close-illusory and far-illusory conditions (Kruskal–Wallis test).

To further identify the areas of activation in the cerebrum, sLORETA (standard low resolution brain electromagnetic tomography) was applied [52]. sLORETA is a methodology to produce a neuro-image of the brain given the measurements of the EEG and to find the 3D distribution of the generating electric neuronal activity. It belongs to a family of linear imaging methods with exact, zero error localization to point-test sources. sLORETA, which indicates activation of the brain from EEG data, is based on a predefined brain anatomy template, the generic MRI set. Although sLORETA does not take brain images of participants for analysis, activation of subjects’ brain mapped to a sLORETA generic brain template is a powerful tool. It has been shown that sLORETA has no localization bias in the presence of measurement and biological noise. In addition to the imaging capability, sLORETA also includes a statistical analysis package, which was applied to compare the reference (veridical) and achieved (illusory) using the paired *t*-test. The differences between the veridical and illusory sensation was analyzed at 8 different frequency bands of the EEG data, namely the delta (1.5–6 Hz), theta (6.5–8 Hz), alpha-1 (8.5–10 Hz), alpha-2 (10.5–12 Hz), beta-1 (12.5–18 Hz), beta-2 (18.5–21 Hz), beta-3 (21.5–30 Hz) and gamma (30.5–50 Hz). The delta band of the EEG has been observed to be related not only to sleep, but also sustainable task performance [53,54,55]. However, attention and memory processes have effects on all of the bands of the EEG [20,21,22,23]. It is necessary to examine all the bands of the EEG in this study to better understand the different patterns of the EEG band according to human perception and cognition.

The median score of the post-experiment questionnaire from participants was calculated, and the correlation of those of Q1 and Q3 and P300 latency for frontal and parietal lobe was analyzed.

## 5. Results

In this section, we describe the results of the ERP and sLORETA analysis and subjective questionnaires.

### 5.1. ERP Result

**Figure 6:**
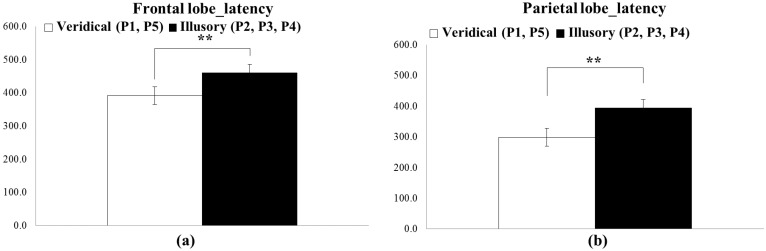
Significant differences shown in the ERP latency between the illusory sensation (at P2, P3 and P4, black box) and the veridical one (at P1 and P5, white box) in: (**a**) frontal lobe; and (**b**) parietal lobe (based on the Mann–Whitney U test) (** *p* < 0.05).

**Figure 7:**
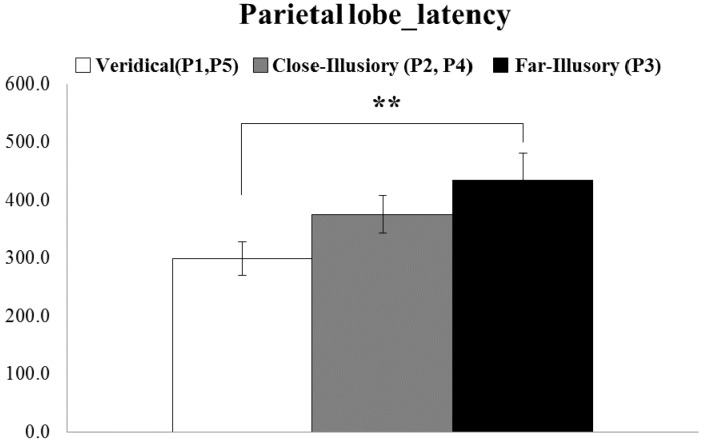
Significant differences shown in the ERP latency between the far-illusory (P3, black box) and veridical (P1 and P5, white box) sensation in all of the parietal lobe (based on the Kruskal-Wallis test) (** *p* < 0.05).

The ERP analysis showed statistically-significant differences in the latency between the illusory and veridical tactile sensation in frontal lobe (Channels Fp1, Fp2, F7, F3, Fz, F4, F8) and parietal lobe (Channels P3, Pz, P4), shown in Figure 6. No other differences were observed among other local brain areas (e.g., temporal, central and occipital). In frontal lobe, the ERP latency for the veridical sensation (at P1 and P5) was, on average, 391 ms, while for the illusory one (at P2, P3 and P4) 460 ms, a statistically-significant difference of 69 ms, as shown in Figure 6a (Z = −1.989, *p* < 0.05, r = −0.33). This, in turn, means that the illusory tactile sensation was a more delayed reaction than the veridical one. In parietal lobe, the latency difference was as much as 134 ms for the P3 alone, as shown in Figure 6b (χ^2^(2, N = 18) = 7.189, *p* < 0.05, Cramér’s V = 0.0771). In addition, when parietal lobe activation was compared in three treatment groups using the Kruskal-Wallis test, the latency for veridical tactile latency (P1 and P5) was 299 ms and for the far-illusory (P3) 433 ms, as shown in Figure 7 (*p* < 0.05). No difference was shown between the close-illusory (at P2, P4) and far-illusory (at P3) conditions. As for ERP amplitudes, no significant effects were found in any of the channels.

5.2. sLORETA Result

**Figure 8:**
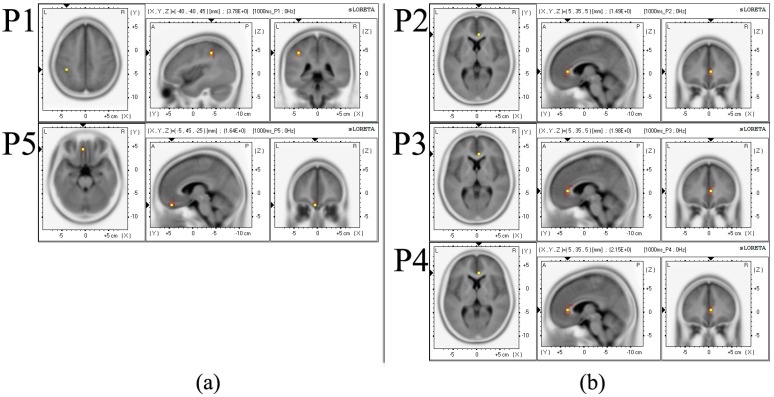
Brain activation areas in the delta band frequency: (**a**) veridical (at P1, P5); and (**b**) illusory (at P2–P4). The activation of a subject mapped in a sLORETA (standard low resolution brain electromagnetic tomography) generic brain template is presented.

The comparative sLORETA results between the reference and acquired data, from all channels during the stimulation, package are shown in Figure 8. To summarize the figure, at P1, the delta band was activated at the inferior parietal lobule of parietal lobe. On the other hand, at P2, P3 and P4, the delta bands were activated at the anterior cingulate of limbic lobe. At P5, the delta band was activated at the orbital gyrus of frontal lobe (also see Table 3). Similarly, Table 3 describes the regional brain activity in all of the discrete frequency bands, and the “*” mark is used to indicate a statistically-significant difference (*p* < 0.05) (between the veridical and illusory sensation) and the existence of distinct brain activity.

**Table 3:** Brain activation areas in discrete frequency bands (* a statistically-significant difference is found between the veridical and illusory sensation).

Location	MNI (Montreal Neurological Institute) Coordinates			tStatistic	Brain Area	Brodmann Area
	x	y	z			
**Delta band**						
P1 *	−40	−40	45	3.776	inferior parietal lobule, parietal lobe	40
P2 *	5	35	5	1.495	anterior cingulate, limbic lobe	24
P3 *	5	35	5	1.978	anterior cingulate, limbic lobe	24
P4 *	5	35	5	2.149	anterior cingulate, limbic lobe	24
P5 *	−5	45	−25	1.639	Orbital gyrus, frontal lobe	11
**Theta band**						
P1	−45	−45	55	2.505	inferior parietal lobule, parietal lobe	40
P2	−55	35	0	0.873	inferior frontal gyrus, frontal lobe	47
P3	10	25	25	1.041	anterior cingulate, limbic lobe	32
P4	−5	35	−25	1.319	rectal gyrus, frontal lobe	11
P5	−55	35	0	1.096	inferior frontal gyrus, frontal lobe	47
**Alpha-1 band**						
P1	−45	−45	55	1.554	inferior parietal lobule, parietal lobe	40
P2 *	−25	35	−5	1.279	inferior frontal gyrus, frontal lobe	47
P3 *	−5	30	−20	1.150	medial frontal gyrus, frontal lobe	25
P4 *	−25	30	−5	1.144	inferior frontal gyrus, frontal lobe	47
P5 *	−45	35	−10	1.302	inferior frontal gyrus, frontal lobe	47
**Alpha-2 band**						
P1	−50	−45	55	1.131	inferior parietal lobule, parietal lobe	40
P2	−5	30	−25	0.548	rectal gyrus, frontal lobe	11
P3	5	50	40	0.800	medial frontal gyrus, frontal lobe	9
P4	5	20	−5	0.650	anterior cingulate, limbic lobe	25
P5	−5	55	40	0.524	medial frontal gyrus, frontal lobe	9
**Beta-1 band**						
P1	−40	−45	45	0.880	inferior parietal lobule, parietal lobe	40
P2	5	25	15	0.586	anterior cingulate, limbic lobe	24
P3	5	−50	70	0.797	postcentral gyrus, limbic lobe	5
P4	5	25	15	0.650	anterior cingulate, limbic lobe	24
P5	5	30	20	0.540	anterior cingulate, limbic lobe	24
**Beta-2 band**						
P1	−35	−50	45	0.512	inferior parietal lobule, parietal lobe	40
P2	−60	−55	70	0.325	middle temporal gyrus, temporal lobe	37
P3	−5	−55	70	0.599	postcentral gyrus, parietal lobe	7
P4	−65	−50	70	0.401	superior temporal gyrus, temporal lobe	22
P5	−60	−65	70	0.336	inferior temporal gyrus, temporal lobe	37
**Beta-3 band**						
P1	−35	−50	45	0.512	inferior parietal lobule, parietal lobe	40
P2	10	−60	70	0.325	postcentral gyrus, parietal lobe	7
P3	5	−50	70	0.599	postcentral gyrus, parietal lobe	5
P4	10	−55	70	0.401	postcentral gyrus, parietal lobe	7
P5	5	−55	70	0.336	postcentral gyrus, parietal lobe	7
**Gamma band**						
P1	−15	−55	60	0.529	superior parietal lobule, parietal lobe	7
P2	−5	−50	60	0.481	precuneus, parietal lobe	7
P3	−5	−50	60	0.576	precuneus, parietal lobe	7
P4	−5	−50	60	0.611	precuneus, parietal lobe	7
P5	−5	−50	55	0.503	precuneus, parietal lobe	7

### 5.3. Subjective Questionnaires

**Figure 9:**
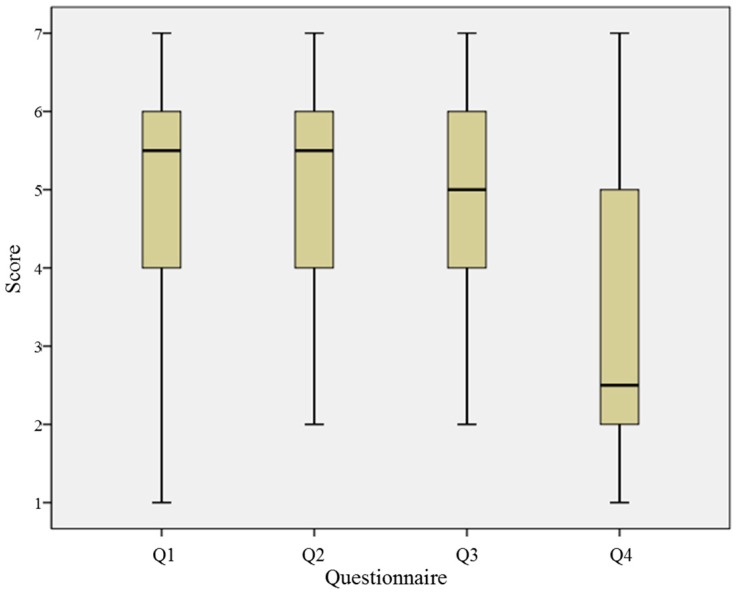
Results of the post-questionnaire.

As already mentioned, subjects were asked the four survey questions shown in Table 1 (Q1: existence of the tactile feedback; Q2: recognition of the stimulation location; Q3: time taken to recognize or perceive the sensation; Q4: certainty of their own answers). The Likert scoring scale was used for the questions (7.0 being the highest scoring point). The scale and contents of the questionnaire are summarized in Table 1. The medians of each questionnaire were 5.5, 5.5, 5.0 and 2.5 in ascending order, respectively (see Figure 9).

The correlation between the result of the post-questionnaire Q1 and Q3 and P300 latency of frontal and parietal lobe was analyzed. The result of Q1 showed a linear relationship with P300 latencies of the frontal and parietal lobe EEG (frontal lobe: *r* = 0.278, *p* > 0.05; parietal lobe: *r* = 0.314, *p* > 0.05). The result of Q3 also did not show a linear relationship with P300 latencies of the frontal and parietal lobe EEG (frontal lobe: *r* = 0.251, *p* > 0.05; parietal lobe: *r* = 0.235, *p* > 0.05).

Regression analysis of P300 latency showed that 7.7% and 9.8% of the frontal and parietal lobe EEG variance can be explained by the result of Q1, respectively (frontal lobe: *r*^2^ = 0.077; parietal lobe: *r*^2^ = 0.098). Same analysis with Q3 showed that 12.8% and 9.7% of the frontal and parietal lobe EEG variance can be explained by the result of the questionnaire (frontal lobe: *r*^2^ = 0.128; parietal lobe: *r*^2^ = 0.097).

## 6. Discussion

The main purpose of the current study was to identify differences in the brain activations between the veridical and illusory tactile sensation, and furthermore, to provide a possible brain-scientific explanation to the out of the body tactile illusion on virtual or real objects. 

ERP latencies for the illusory and veridical tactile showed prominent distinctions. For example, when the veridical tactile (P1 and P5) and illusory tactile (P2, P3, and P4) sensation were compared in two separate groups, significant differences were found in the parietal and frontal area. When compared in three groups (namely veridical, close-illusory and far-illusory), the differences in P3 latency were observed in parietal lobe; only between the veridical and far-illusory.

The neuronal pathway of tactile information was reported to follow the “where” information (spatial information) pathway in somatosensory cortex of parietal lobe and then pass the working memory pathway located on dorsolateral prefrontal cortex [56,57,58,59]. As Figure 7 shows, the P300 latency of veridical and illusory information was significantly different only at parietal and frontal lobe. These findings suggest the possibility that the response of illusory information imposes a cognitive load, which may have resulted in slower propagation of the EEG along the pathway. The ERP when cross-modal tactile sensation with visual feedback was present showed a difference with that observed while uni-modal tactile sensation was present [60]. Its delay might have occurred due to memory process of past tactile experience [61].

Alles [7] has observed localized tactile phantom sensations according to different stimulation intensities. Our experiment also showed, in terms of the ERP latencies, significant differences between the veridical (P1, P5) and far-illusory (P3) sensation.

The EEG response results showed that parietal/frontal lobes were mainly activated for the veridical and limbic lobe for the illusory sensation, only in the delta band frequency. Interestingly, parietal lobe (somatosensory area) is known to be heavily associated with tactile perception, while limbic lobe with memory, cognition and perceptual illusions [62,63,64].

At low-frequency bands (1.5–12 Hz: delta, theta, alpha-1 and alpha-2), generally (even though some results do not have statistically-significant differences), stimulation at P1 (veridical) activated parietal lobe. Stimulations at P2, P3 and P4 (illusory) activated limbic and frontal lobe. At mid-frequency bands (12.5–21 Hz: beta-1 and beta-2), P1 (veridical) and P3 (illusory) activated parietal lobe. P2 and P4 (illusory) activated limbic and frontal lobe. At high-frequency bands (21.5–50 Hz: beta-3 and gamma), P1–P5 all activated parietal lobe. To summarize, grossly, parietal lobe was involved in both the veridical and illusory sensation, while limbic lobe only appeared in the illusory sensation. Again, overall, the activation of parietal lobe is consistent with prior studies in the brain pathways for tactile information processing [65].

Note that in the delta band, the veridical stimulations on the left fingertip, P1, activated inferior parietal lobule (Brodmann area 40 (BA 40) in the somatosensory area (especially, secondary somatosensory cortex, SII) in the left hemisphere. In particular, the secondary somatosensory cortex (SII) includes parts of BA40 and 43 [66]. Reed *et al.* [65] have reported that activation of SII along with insula, BA40 and parietal association areas played an important role in tactile object recognition. Milner *et al.* [67] suggested that BA40, the somatosensory association area, such as SII, corresponded to the integration of somatosensory information. Additionally, they have reported that these areas carried high-level processing, such as integrating tactile and proprioceptive information. Furthermore, recently, several studies have reported that the parietal region, especially inferior parietal lobule, has relevance to the somatosensory spatial discrimination [68].

In our study, the veridical stimulations on the right fingertip, P5, activated orbital gyrus (BA11) of frontal lobe in contrast with P1. In other words, stimulation at P5 did not activate the somatosensory area like P1. BA11 is a region including orbitofrontal cortex (OFC), where multisensory representation is reconstructed from the output of unimodal processed streams [69]. As multimodal stimulus was presented during the experiment, it was assumed that orbitofrontal cortex was activated.

Then, the funneling stimulation to elicit illusory sensation, that is at P2, P3 and P4, activated the anterior cingulate (BA24) in the limbic lobe. Craig *et al.* [70] have reported that this area was activated by harmful heat or cold tactile stimulation. Devinsky *et al.* [71] have also reported this region was divided into separate areas; affect and cognition divisions, including BA 24. Furthermore, Bäckman *et al.* [38] have found the increase of blood flow in anterior cingulate gyrus during memory recall tasks. Therefore, our results suggest that tactile illusion is associated memory recall and cognition requiring more processing time for recognition in illusory sensation. These reports that the brain region activated by illusory sensation is associated with cognition and memory recall are in line with the P300 latency difference between illusory and veridical sensation on frontal and parietal lobe.

## 7. Conclusions

In this paper, we conducted an EEG measurement and brain imaging analysis for out of the body tactile illusion phenomenon, attempting to gain a glance into the underlying mechanism and hoping to apply it for HCI purposes, such as devising new types of tactile feedback. The experimental data and analysis indicate that both veridical and illusory sensations involve, among others, parietal lobe, one of the most important components in the tactile information pathway. This is despite the lack of any corresponding area in the body map for an external hand-held object, the localization source of the out of the body sensation. In addition, we find that as for the illusory sensation, there is an additional processing resulting in the delay for the ERP and involvement by the limbic lobe. This alludes to regarding illusion as a memory and recognition task. The implication to HCI is that, for example, humans might be conjectured to recognizing only 7–8 illusory locations due to the limitation in working memory [72]. Furthermore, the projected proportionality between the ERP latency and the illusory location interval might place a limit on the maximum distance to which the phenomenon can be applied (e.g., the size of the mobile device or virtual object on which the illusion technique is applied). Certainly, more experiments, analyses and cross-checking with other related work are needed to validate our conjectures.

## References

[1] Borst C.W., Cavanaugh C.D. (2004). Haptic Controller Design and Palm-sized Vibrotactile Array.

[2] Borst C.W., Baiyya V.B. Enhancing VR-based Visualization with a 2D Vibrotactile Array. Proceedings of the 2007 ACM Symposium on Virtual Reality Software and Technology.

[3] Yang G., Jin M., Jin Y., Kang S. T-mobile: Vibrotactile Display pad with Spatial and Directional Information for Hand-held Device. Proceedings of the Intelligent Robots and Systems (IROS), 2010 IEEE/RSJ International Conference on Taipei International Convention Center.

[4] Piateski E., Jones L. Vibrotactile Pattern Recognition on the Arm and Torso. Proceedings of the First JointEurohaptics Conferenceand Symposium on Haptic Interfaces for Virtual Environment and Teleoperator Systems, World Haptics 2005.

[5] Geldard F.A., Sherrick C.E. (1972). The Cutaneous “Rabbit”: A Perceptual Illusion. Science.

[6] Flach R., Haggard P. (2006). The cutaneous rabbit revisited. J. Exp. Psychol.Hum. Percept. Perform..

[7] Alles D.S. (1970). Information transmission by phantom sensations. IEEE Trans. Man-Mach. Syst..

[8] Chen L.M., Friedman R.M., Roe A.W. (2003). Optical imaging of a tactile illusion in area 3b of the primary somatosensory cortex. Science.

[9] Gardner E.P., Spencer W.A. (1972). Sensory funneling. I. Psychophysical observations of human subjects and responses of cutaneous mechanoreceptive afferents in the cat to patterned skin stimuli. J. Neurophysiol..

[10] Miyazaki M., Hirashima M., Nozaki D. (2010). The “cutaneous rabbit” hopping out of the body. J. Neurosci..

[11] Eimer M., Forster B., Vibell J. (2005). Cutaneous saltation within and across arms: A new measure of the saltation illusion in somatosensation. Percept. Psychophys..

[12] Kim Y., Lee J., Kim G. Extending out of the Body Saltation to 2D Mobile Tactile Interaction. Proceedings of the 10th asia pacific conference on Computer human interaction.

[13] Lipari N.G., Borst C.W. (2014). Study of 2D Vibration Summing for Improved Intensity Control in Vibrotactile Array Rendering. Adv. Vis. Comput..

[14] Lee J., Kim Y., Kim G. Funneling and Saltation Effects for Tactile Interaction with Virtual Objects. Proceedings of the ACM SIGCHI Conference on Human Factors in Computing Systems.

[15] Chen L.M., Turner G.H., Friedman R.M., Zhang N., Gore J.C., Roe A.W., Avison M.J. (2007). High-resolution maps of real and illusory tactile activation in primary somatosensory cortex in individual monkeys with functional magnetic resonance imaging and optical imaging. J. Neurosci..

[16] Chen L.M., Friedman R., Roe A.W. (2010). Somatosensory: Imaging Tactile Perception. Imaging the Brain with Optical Methods.

[17] Gross L. (2006). Classic illusion sheds new light on the neural site of tactile perception. PLoS Biol..

[18] Cohen M.J., Ament P.A., Schandler S.L., Vulpe M. (1996). Changes in the P300 component of the tactile event-related potential following spinal cord injury. Spinal Cord.

[19] Mitsudo T., Gagnon C., Takeichi H., Grondin S. (2011). An electroencephalographic investigation of the filled-duration illusion. Front. Integr. Neurosci..

[20] Petsche H., Pockberger H., Rappelsberger P. (1986). EEG Topography and Mental Performance. Topographic Mapping of Brain Electrical Activity.

[21] Sauseng P., Klimesch W., Stadler W., Schabus M., Doppelmayr M., Hanslmayr S., Birbaumer N. (2005). A shift of visual spatial attention is selectively associated with human EEG alpha activity. Eur. J. Neurosci..

[22] Gevins A.S., Zeitlin G.M., Doyle J.C., Yingling C.D., Schaffer R.E., Callaway E., Yeager C.L. (1979). Electroencephalogram correlates of higher cortical functions. Science.

[23] Jensen O., Kaiser J., Lachaux J.P. (2007). Human gamma-frequency oscillations associated with attention and memory. Trends Neurosci..

[24] Valentino D.A., Arruda J.E., Gold S.M. (1993). Comparison of QEEG and response accuracy in good vs poorer performers during a vigilance task. Int. J. Psychophysiol..

[25] Bekesy G.V. (1958). Funneling in the nervous system and its role in loudness and sensation intensity on the skin. J. Acoust. Soc. Am..

[26] Barghout A., Cha J., el Saddik A., Kammerl J., Steinbach E. Spatial Resolution of Vibrotactile Perception on the Human Forearm when Exploiting Funneling Illusion. Proceedings of the IEEE International Workshop on Haptic Audio visual Environments and Games.

[27] Rahal L., Cha J., El Saddik A., Kammerl J., Steinbach E. Investigating the Influence of Temporal Intensity Changes on Apparent Movement Phenomenon. Proceedings of the 2009 IEEE International Conference on Virtual Environments, Human-Computer Interfaces and Measurement Systems (VECIMS).

[28] Mizukami Y., Sawada H. (2006). Tactile information transmission by apparent movement phenomenon using shape-memory alloy device. Int. J. Disabil. Hum. Dev..

[29] Tan H.Z., Pentland A. (1997). Tactual displays for wearable computing. Pers. Technol..

[30] Ramachandran V.S., Blakeslee S., Sacks O.W. (1998). Phantoms in the Brain: Probing the Mysteries of the Human Mind.

[31] Bark K., Hyman E., Tan F., Cha E., Jax S.A., Buxbaum L.J., Kuchenbecker K.J. (2014). Effects of Vibrotactile Feedback on Human Learning of Arm Motions. IEEE Trans. Neural Syst. Rehabil. Eng..

[32] Ma K., Hommel B. (2014). The virtual-hand illusion: Effects of impact and threat on perceived ownership and affective resonance. Front. Psychol..

[33] Ehrsson H.H., Holmes N.P., Passingham R.E. (2005). Touching a rubber hand: feeling of body ownership is associated with activity in multisensory brain areas. J. Neurosci..

[34] Slater M., Perez-Marcos D., Ehrsson H.H., Sanchez-Vives M.V. (2008). Towards a digital body: The virtual arm illusion. Front. Hum. Neurosci..

[35] Sanchez-Vives M.V., Spanlang B., Frisoli A., Bergamasco M., Slater M. (2010). Virtual hand illusion induced by visuomotor correlations. PLoS ONE.

[36] Padilla-Castañeda M.A., Frisoli A., Pabon S., Bergamasco M. (2014). The modulation of ownership and agency in the virtual hand illusion under visuotactile and visuomotor sensory feedback. Presence.

[37] Blankenburg F., Ruff C.C., Deichmann R., Rees G., Driver J. (2006). The cutaneous rabbit illusion affects human primary sensory cortex somatotopically. PLoS Biol..

[38] Bäckman L., Almkvist O., Andersson J., Nordberg A., Winblad B., Reineck R., Långström B. (1997). Brain activation in young and older adults during implicit and explicit retrieval. J. cogn. neurosci..

[39] Shared Space/ARToolKit Download Page. http://www.hitl.washington.edu/ARToolKit/.

[40] Arduino. http://www.arduino.cc/.

[41] Jahwa Electronics. http://www.jahwa.co.kr/.

[42] Jung J., Choi S. Perceived Magnitude and Power Consumption of Vibration Feedback in Mobile Devices. Proceedings of the 12th International Conference on Human-Computer Interaction. Interaction Platforms and Techniques.

[43] Burdea G., Coiffet P. (2003). Virtual Reality Technology.

[44] Sherrick C.E. (1985). A scale for rate of tactual vibration. J. Acoust. Soc. Am..

[45] Jasper H.H. (1958). The ten twenty electrode system of the international federation. Electroencephalogr. Clin. Neurophysiol..

[46] Jolliffe I.T. (2002). Principal Component Analysis (Springer Series in Statistics).

[47] Carter R. (2014). The Human Brain Book.

[48] Ros T., Théberge J., Frewen P.A., Kluetsch R., Densmore M., Calhoun V.D., Lanius R.A. (2013). Mind over chatter: Plastic up-regulation of the fMRI salience network directly after EEG neurofeedback. Neuroimage.

[49] Zaehle T., Sandmann P., Thorne J.D., Jäncke L., Herrmann C.S. (2011). Transcranial direct current stimulation of the prefrontal cortex modulates working memory performance: Combined behavioural and electrophysiological evidence. BMC Neurosci..

[50] Zhuang X., Sekiyama K., Fukuda T. Evaluation of Human Sense by Biological Information Analysis. Proceedings of the IEEE International Symposium on Micro-NanoMechatronics and Human Science (MHS).

[51] Brouwer A.M., van Erp J.B. (2010). A tactile P300 brain-computer interface. Front. Neurosci..

[52] Pascual-Marqui R. (2002). Standardized low-resolution brain electromagnetic tomography (sLORETA): Technical details. Methods Find. Exp. Clin. Pharmacol..

[53] Dolce G., Waldeier H. (1974). Spectral and multivariate analysis of EEG changes during mental activity in man. Electroen. Clin. Neurosci..

[54] Kakizaki T. (1985). Evaluation of mental task strain based on occipital beta activity and subjective rating of task difficulty. Eur. J. Appl. Physiol..

[55] Tucker D.M., Dawson S.L., Roth D.L., Penland J.G. (1985). Regional changes in EEG power and coherence during cognition: Intensive study of two individuals. Behav. neurosci..

[56] Cohen D. (1996). Secret Language of the Mind: A Visual Inquiry Into the Mysteries of Consciousness.

[57] Collins M.L., Nelson C.A., Luciana M., Nelson C.A., Luciana M. (2001). Handbook of Developmental Cognitive Neuroscience.

[58] Funahashi S., Bruce C.J., Goldman-Rakic P.S. (1989). Mnemonic coding of visual space in the monkey’s dorsolateral prefrontal cortex. J. Neurophysiol..

[59] Smith E.E., Jonides J. (1999). Storage and executive processes in the frontal lobes. Science.

[60] Ohara S., Lenz F., Zhou Y. (2006). Sequential neural processes of tactile–visual crossmodal working memory. Neuroscience.

[61] Fuster J.M. (2001). The prefrontal cortex—An update: Time is of the essence. Neuron.

[62] Carter C.S., Botvinick M.M., Cohen J.D. (1999). The contribution of the anterior cingulate cortex to executive processes in cognition. Rev. Neurosci..

[63] Bachevalier J., Brickson M., Hagger C. (1993). Limbic-dependent recognition memory in monkeys develops early in infancy. Neuroreport.

[64] Gloor P., Olivier A., Quesney L.F., Andermann F., Horowitz S. (1982). The role of the limbic system in experiential phenomena of temporal lobe epilepsy. Ann. Neurol..

[65] Reed C.L., Shoham S., Halgren E. (2004). Neural substrates of tactile object recognition: An fMRI study. Hum. Brain. Map..

[66] Benarroch E.E. (2006). Basic Neurosciences with Clinical Applications.

[67] Milner T.E., Franklin D.W., Imamizu H., Kawato M. (2007). Central control of grasp: Manipulation of objects with complex and simple dynamics. NeuroImage.

[68] Akatsuka K., Noguchi Y., Harada T., Sadato N., Kakigi R. (2008). Neural codes for somatosensory two-point discrimination in inferior parietal lobule: An fMRI study. NeuroImage.

[69] ROLLS E.T., Stein B.E. (2004). 19. Multisensory Neuronal Convergence of Taste, Somatosensory, Visual, Olfactory, and Auditory Inputs. The Handbook of Multisensory Processes.

[70] Craig A.D., Reiman E.M., Evans A., Bushnell M.C. (1996). Functional imaging of an illusion of pain. Nature.

[71] Devinsky O., Morrell M.J., Vogt B.A. (1995). REVIEW ARTICLE Contributions of anterior cingulate cortex to behavior. Brain.

[72] Miller G.A. (1956). The magical number seven, plus or minus two: Some limits on our capacity for processing information. Psychol. Rev..

